# Expression changes of autophagy-related proteins in AKI patients treated with CRRT and their effects on prognosis of adult and elderly patients

**DOI:** 10.1186/s12979-018-0128-5

**Published:** 2018-10-03

**Authors:** Yang Zhang, Ling Wang, Lei Meng, Guang-Ke Cao, Yu-Liang Zhao, Yu Wu

**Affiliations:** 1 Department of Anesthesiology, Xuzhou Medical University, Xuzhou, 221000 Jiangsu China; 2 Department of Nephrology, The First People’s Hospital of Xuzhou, Xuzhou Municipal Hospital Affiliated to Xuzhou Medical University, Xuzhou, Jiangsu 221000 People’s Republic of China

**Keywords:** AKI, CRRT, LC3-II, Atg-5, Beclin-1, Prognosis

## Abstract

**Background:**

Sepsis is one of the common death factors in intensive care unit, which refers to the systemic inflammatory response syndrome caused by infection. It has many complications such as acute renal injury, shock, multiple organ dysfunction, and failure. The mortality of acute renal injury is the highest among the complications, which is a serious threat to the safety of patients and affects the quality of life. This study aimed to observe the changes in autophagy-related protein expressions in patients with acute kidney injury (AKI) after continuous renal replacement therapy (CRRT) and their impacts on prognosis.

**Methods:**

207 AKI patients visiting the Emergency Department of The First People’s Hospital of Xuzhou from January 2014 to February 2018 were recruited and treated with CRRT. Quantitative reverse transcription polymerase chain reaction (qRT-PCR) was applied to detect the expression of autophagy-related genes, including light chain 3 type II (LC3-II), autophagy-related 5 (Atg-5) and Beclin-1, in the monocytes of the patient’s peripheral blood before and after treatment. The levels of inflammatory mediators interleukin (IL)-1β and IL-6 were determined via enzyme-linked immunosorbent assay before and after treatment. The patient’s serum creatinine (Scr) level before and after treatment was measured using a full-automatic biochemistry analyser. Moreover, the treatment effect was graded after CRRT, and the relationship between the prognosis of patients and the autophagy-related proteins was observed.

**Results:**

The Scr levels in the patients were significantly decreased after treatment with CRRT. Before treatment, the IL-1β and IL-6 blood levels were high in the patients, while the amounts were significantly reduced after CRTT. The expressions of LC3-II, Atg-5 and Beclin-1 in the monocytes of patients after treatment were significantly decreased compared with those before treatment. Compared with those in survived patients, the expression of autophagy-related proteins was significantly elevated in in patients died after one to three weeks after the treatment. IL-1β, IL-6, LC3-II and Beclin-1, but not Atg-5 values were significantly correlated with Scr.

**Conclusion:**

The expression of LC3-II, Atg-5 and Beclin-1 in the monocytes of patients may change prominently after treatment with CRRT, so they are expected to be regarded as new prognostic indicators for AKI patients.

## Background

Acute kidney injury (AKI) is defined as an abrupt decrease in kidney function, which encompasses structural damage and impairment loss of function of kidney. The prognosis of AKI patients is far from satisfactory, and surveys show that the mortality of AKI patients in intensive care unit may be up to 50%. However, 20% of the surviving patients still have chronic renal insufficiency after treatment, which can ultimately develop into end-stage renal disease, so the repeated renal replacement therapy is required. It poses a serious threat to the life safety and quality of the patients, but there are always some controversies over the AKI prognostic indicators [1–3].

AKI has been shown to be closely related to autophagy, which degrades and removes damaged proteins and aged organelles through the mediation of lysosomes, hence maintaining the stability of internal environment in the body [4–6].

Currently, studies on autophagy-related proteins mainly focus on light chain 3 (LC3), autophagy-related 5 (Atg-5), and Beclin-1. During autophagy, cytoplasmic components, including cytosolic proteins and organelles are engulfed by autophagosomes. In the mean time, a cytosolic form of LC3 (LC3-I) is conjugated to phosphatidylethanolamine to form LC3-phosphatidylethanolamine conjugate (LC3-II), which is recruited to autophagosomal membranes. Atg-5, produced by autophagic vesicles, is an important protein for extension of phagocytic membrane. Beclin-1 is a key protein that regulates autophagy and cell death, and studies have revealed that Beclin-1 is differentially expressed in the renal tissues of rat models of renal ischemia-reperfusion injury [4–8].

The differential levels of pro-inflammatory cytokines interleukin (IL)-1β and IL-6, representatives of the pro-inflammatory cytokines, well reflect the degree of inflammatory responses in the body [9]. Serum creatinine (Scr) is regarded as a crucial index manifesting the health of human kidney because it is a by-product of muscle metabolism that can be easily measured. Moreover, it can act as one of the indexes reflecting the situations of renal function, since it is filtered by kidney glomeruli and is entirely eliminated in the urine, without being reabsorbed at the tubular level [10].

Continuous renal replacement therapy (CRRT) exerts prominent application effects in first aid of AKI and plays vital roles in stabilizing hemodynamic and internal environment of the body [11].

Therefore, in this study, the expressions of LC3-II, Atg-5, Beclin-1 in monocytes, and the levels of IL-1β, IL-6 and Scr in the peripheral blood of AKI patients were performed to analyse the changes in these indexes before and after treatment with CRRT, with the aim to find novel indexes for clinical prognosis.

## Methods

### Patients

In this research, a total of 207 AKI patients attending the Emergency Department of The First People’s Hospital of Xuzhou from January 2014 to February 2018 were selected, including 124 males and 83 females aged 28–75 years old, with an average age of 59.8 ± 8.4 years old. All the AKI patients received CRRT. The patients were divided into the survival group (*n* = 157) and the death group (*n* = 50) according to the survival of the patients. Most of the deaths were caused by multiple organ failure, of which 5 patients died of two-organ failure, 9 patients died of three-organ failure, 28 patients died of four-organ failure, and 8 patients died of five-organ failure. All the 50 patients died after one to three weeks after the study. This research was approved by the Medical Ethics Committee of The First People’s Hospital of Xuzhou, and the patients and their families were informed of the research and signed the informed consent.

Inclusion criteria were: 1) Patients aged ≥ 28 years old, 2) patients conforming to the Clinical Practice Guideline for AKI published by the Kidney Disease: Improving Global Outcomes [12], 3) patients having no blood relations with each other, 4) patients with no history of taking nephrotoxic drugs and 5) patients without liver cirrhosis, autoimmune disease and depression.

Exclusion criteria were: 1) Patients who underwent renal transplantation and regular dialysis before treatment, 2) patients with a course of disease shorter than half a year 3) patients unwilling to cooperate with the treatment and follow-up, and 4) patients with incomplete clinical data.

### Therapeutic methods for patients

All the 207 patients were treated with CRRT, and continuous blood purification was performed for the patients before treatment. After that, the position of venous catheter was selected according to the disease conditions and clinical situations of the patients. Continuous veno-venous hemodiafiltration methods were applied to purify the patients blood, and continuous ultrafiltration ≥2 days was conducted, with a time interval of 1–1.5 day. Post-dilution infusion was conducted using replacement fluid [3000 mL normal saline + 500 mL sterile water + 20 mL 50% glucose solution (GS) + 3.2 mL 25% MgSO_4_ + 35 mL 10% calcium gluconate + 10 mL 10% KCl], the calcium preparation for the patients was adjusted in accordance with the examination findings, and the KCl in the replacement fluid was complemented in time. The death status of the patients at 28 days after treatment was observed.

### Enzyme-linked immunosorbent assay (ELISA) methods and Scr detection

Before and after treatment, 5 mL fasting venous blood was collected from the patients to detect the amounts of IL-1β (Beyotime Institute of Biotechnology) and IL-6 (Beyotime Institute of Biotechnology) according to manufacturer instructions. The Scr in the patients was measured through Hitachi 7600P full-automatic biochemistry analyser.

### Separation of monocytes from the peripheral blood

A total of 5 mL fasting peripheral blood was drawn from the patients before and after treatment, of which cells were isolated by virtue of lymphocyte separating medium. After that, density gradient centrifugation and separation were conducted to collect the monocytes of the peripheral blood, followed by washing, purification and cell count. The trypan blue was utilized to detect the cell viability (the cell viability was required to be over 95%), and then cells were stored at − 80 °C until use.

### Detection via quantitative reverse transcription polymerase chain reaction (qRT-PCR)

TRIzol reagent (Invitrogen, Termo Fisher, Shanghai, China) was applied to lyse the monocytes and extract total ribonucleic acid (RNA), which was reversely transcribed into complementary deoxyribonucleic acid (cDNA) according to the instructions of the reverse transcription kit (Invitrogen). Then PCR amplification was performed with cDNA as the template, and the amplification primers were designed and synthesized by Sangon Biotech (Shanghai, China) Co., Ltd. (Table 1). The PCR kit was purchased from Invitrogen, and the PCR system contained 10 μL Maxima ™ SYBR Green qPCR Master Mix (2X), 200 μmol/L deoxyribonucleoside triphosphates (dNTPs), 20 pmol forward primer, 20 pmol reverse primer, 0.5 μg DNA template and double distilled water complementing the system to 20 μL. PCR conditions: pre-denaturation at 94 °C for 1 min and 40 cycles of 94 °C for 30 s, 55 °C for 30 s and 72 °C for 30 s. The β-actin was used as the internal reference gene in this experiment, which was performed for 3 times in total. The relative expression level was calculated using the 2^-ΔCt^ method [http://www.protocol-online.org/biology-forums-2/posts/26662.html].

**Table 1:** Primer sequences

Gene	Forward primer	Reverse primer
LC3-II	5’-AACATGAGCGAGTTGGTCAAG-3’	5’-GCTCGTAGATGTCCGCGAT-3’
Atg-5	5’-TGACCAGTTTTGGACCATCA-3’	5’-AGGGTATGCAGCTGTCCATC-3’
Beclin-1	5’-AACCAACGTCTTTAATGCAACCTTC-3’	5’-AGCAGCATTAATCTCATTCCATTCC-3’
β-actin	5’-TGGCACCCAGCACAATGAA-3’	5’-CTAAGTCATAGTCCGCCTAGAAGCA-3’

The amplification primers were designed and synthesized by Sangon Biotech (Shanghai, China) Co., Ltd.

### Statistical analysis

Statistical Product and Service Solutions (SPSS) software package (Shanghai Cabit Information Technology Co., Ltd.) was adopted to analyse the collected data, and GraphPad Prism 5 software package (SOFTHEAD) was utilized to draw pictures. The measurement data were presented as mean ± standard deviation (Mean ± SD) and analysed with paired *t*-test. Pearson correlation analysis was performed for various indexes. *P* < 0.05 suggested that the difference was statistically significant.

## Results

### Blood levels of Scr, IL-1β and IL-6 of the patients before and after treatment

The levels of Scr, IL-1β and IL-6 in the serum of the AKI patients were analysed. The Scr levels of the patients after treatment with CRRT [(268.4 ± 98.5) μmol/L] were significantly (*p* < 0.001) lower than that before treatment [(421.9 ± 133.5) μmol/L]. The levels of IL-1β and IL-6 in the serum were measured by ELISA, and, after treatment, the levels of IL-1β [(248.5 ± 44.7) pg/mL] and IL-6 [(25.7 ± 4.1) μg/L] were decreased compared with those before treatment [(345.8 ± 55.4) pg/mL and (38.4 ± 6.2) μg/L] (*p* < 0.001; *p* < 0.001) (Table 2).

**Table 2:** Comparisons of serum levels of Scr, IL-1β and IL-6 of the patients before and after treatment with CRRT

	Before treatment	After treatment	*p*
Scr (μmol/L)	421.9 ± 133.5	268.4 ± 98.5	< 0.001
IL-1β (pg/mL)	345.8 ± 55.4	248.5 ± 44.7	< 0.001
IL-6 (μg/L)	38.4 ± 6.2	25.7 ± 4.1	< 0.001

For further explanation see text

### Expression of autophagy-related genes of the patients before and after treatment

**Fig. 1:**
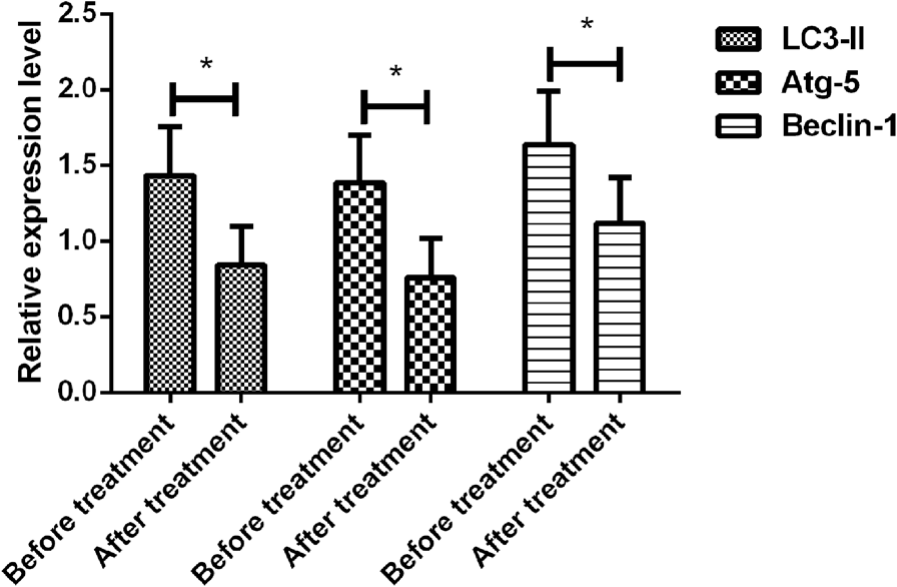
Expression of autophagy-related genes LC3-II, Atg-5 and Beclin-1 of the patients before and after treatment with CRTT. The detection results of LC3-II, Atg-5 and Beclin-1 expression in the monocytes of peripheral blood by means of qRT-PCR show that the relative expression level of LC3-II after treatment is different from that before treatment, which is lowered significantly (*p* = 0.001). There is a difference in Atg-5 relative expression level before and after treatment, and the level is decreased remarkably after treatment (*p* = 0.001). Compared with that before treatment, the relative expression level of Beclin-1 is reduced notably, with a statistical difference (*p* = 0.001)

The expressions of autophagy-related proteins, i.e., LC3-II, Atg-5 and Beclin-1, in the monocytes of the patients peripheral blood were detected by means of qRT-PCR. The expression of autophagy-related proteins LC3-II, Atg-5 and Beclin-1 in the patients after treatment was significantly decreased compared with those before treatment (*p* < 0.001) (Fig. 1).

### Expressions of autophagy-related genes of the survived and died patients after one to three weeks after the treatment

LC3-II, Atg-5 and Beclin-1 expressions measured through qRT-PCR in the monocytes showed that the expressions of autophagy-related proteins (LC3-II, Atg-5 and Beclin-1) were significantly (*p* < 0.001) elevated in patients died after one to three weeks after the treatment when compared to survived patients (Table 3).

**Table 3:** Expressions of autophagy-related genes of the survived and patients dead within 28 days after treatment

	Survived patient (*n* = 197)	Dead patient (*n* = 50)	*p*
LC3-II	0.93 ± 0.22	1.32 ± 0.15	< 0.001
Atg-5	0.88 ± 0.29	1.20 ± 0.12	< 0.001
Beclin-1	1.02 ± 0.31	1.55 ± 0.16	< 0.001

LC3-II, Atg-5 and Beclin-1 expressions measured through qRT-PCR in the monocytes showed that the expressions of autophagy-related proteins were significantly elevated in patients died after one to three weeks after the treatment when compared to survived patients

### Correlations of IL-1β, IL-6 and autophagy-related proteins with Scr

Pearson correlation analysis was performed for the associations of IL-1β, IL-6 and autophagy-related proteins with Scr. IL-1β, IL-6, LC3-II and Beclin-1 were positively correlated with Scr, displaying statistically significant differences (*p* < 0.05), but Atg-5 had no association with Scr (Table 4).

**Table 4:** Correlations of IL-1β, IL-6 and autophagy-related gene expression with Scr

Group	*R*	*p*
IL-1β	0.84	< 0.05
IL-6	0.72	< 0.05
LC3-II	0.58	< 0.05
Atg-5	0.60	Not Significant
Beclin-1	0.62	< 0.05

Pearson correlation analysis was performed for the associations of IL-1β, IL-6 and autophagy-related proteins with serum levels Scr. IL-1β, IL-6, LC3-II and Beclin-1 were positively correlated with serum Scr, displaying statistically significant differences (*p* < 0.05), but Atg-5 had no association with Scr

## Discussion

In spite of multiple factors for AKI occurrence, sepsis has been suggested to be the primary factor for AKI onset. The specific pathogenesis of AKI has not yet been clarified, and there is no satisfactory standard for the diagnosis and prognosis of the disease. Thus, this is the main reason why a relatively better treatment protocol of AKI cannot be formulated. It has been shown that the endotoxins and inflammatory factors in the body are activated in the process of sepsis onset, thus leading to cell autophagy and inducing type II cell apoptosis. However, regulating the autophagy in the renal tissues is expected to become a method for treating and preventing AKI [1, 3, 4, 6, 11–13].

It was initially believed that autophagy enabled the cells to survive in severe environment, obtaining energy by phagocytizing the cytoplasm and organelle of the autologous cells. However, autophagy can mediate the progression of a variety of cell cases. Moreover, cell autophagy under different circumstances may induce aggregation of massive autophagosomes and deletion of organelles and cytoplasm, thus leading to cell death. Such a process is known as type II programmed cell death. There are several diseases involving autophagy, including heart failure, inflammation and cancer. In the kidney, however, autophagy exerts crucial effects in maintaining physiological functions and protecting renal tubular epithelial cells from injury [4–8, 14].

The expression of LC3-II, a vital autophagy-related protein, can be considered as a marker of autophagy occurrence. The mass accumulation of LC3-II can activate inflammasome of macrophages, apoptosis-associated speck-like protein containing a CARD (ASC), caspase-1 as well as NACHT, LRR and PYD domains-containing protein 3 (NLRP3), thereby releasing a large amount of inflammatory IL-1β, producing plenty of IL-6 and tumor necrosis factor (TNF)-α, so accelerating inflammatory response process and ultimately resulting in aggravated inflammatory injury of the patients. As a specific autophagy-related protein, Beclin-1 has become a marker for detecting autophagic activity. Beclin-1 is a component of phosphatidylinositol 3-kinase (PI3K) complex, which can participate in the construction of autophagosome, and acts as a platform for regulation of Atg protein and complex, promoting the occurrence of autophagy. Being an indispensable gene in the Atg family, Atg-5 can regulate the extension of autophagosome, and its expression change can better reflect the degree of autophagy [14–20].

In our study, the relative expressions of LC3-II, Beclin-1 and Atg-5 in the monocytes of peripheral blood as well as Scr, IL-1β and IL-6 in the serum of the AKI patients were detected. The results indicated that the expressions of all the indexes were decreased after treatment with CRRT, and there were differences in comparison with those before treatment, suggesting that the expressions of LC3-II, Beclin-1 and Atg-5 of the patients are suppressed after CRRT. It is speculated that the occurrence of autophagy is reduced because the CRRT may eliminate inflammatory mediators in the blood and decrease the deletion of cytoplasm and necrosis of organelle. The renal metabolism is improved, and the Scr expression level is lowered due to the decreased autophagy. Accordingly, the expressions of LC3-II, Beclin-1 and Atg-5 in the monocytes of peripheral blood in patients dead within 28 d after treatment were shown to be significantly higher than those observed in survived patients. So the autophagy in dead patients is more severe than that in survived patients.

Finally, the relations of IL-1β, IL-6 and autophagy-related proteins with Scr were analysed using Pearson correlation analysis, and the results revealed that IL-1β, IL-6, LC3-II and Beclin-1 had positive correlations with Scr expression, indicating that those indexes may be related to Scr, index of functional metabolism in the kidney [10, 21].

Nevertheless, there are some limitations in this research. There were few detection time points, so the detailed changes in the autophagy-related proteins in the patients blood during 28 days were not analysed. This is an observational study, so we can only hypothesize the relationship between autophagy and serological markers and do not demonstrate a cause effect relationship. Moreover, a small number of inflammatory indexes were examined in this experiment, and inflammatory factors other than IL-1β and IL-6 were not measured. Therefore, it is necessary to increase the frequency and time of detection in future studies, thus providing more evidence for this research and obtaining better results.

## Conclusion

The expressions of LC3-II, Atg-5 and Beclin-1 in the monocytes of the patients peripheral blood are changed prominently after treatment with CRRT, so they are expected to be regarded as new prognostic indicators for AKI patients.

## Abbreviations

AKI: Acute kidney injury
Atg-5: Autophagy-related 5
CCRT: Continuous renal replacement therapy circumference
cDNA: Complementary deoxyribonucleic acid
dNTPs: Deoxyribonucleoside triphosphates
ELISA: Enzyme-linked immunosorbent assay
GS: Glucose solution
IL: Interleukin
LC3: Light chain 3
LC3-I: Light chain 3 type I
LC3-II: Light chain 3 type II
LRR: Leucine rich repeat
NLRP3: Domains-containing protein 3
PI3K: Phosphatidylinositol 3-kinase;
PYD: Pyridinoline
qRT-PCR: Quantitative reverse transcription polymerase chain reaction
RNAm: Messenger ribonucleic acid
Scr: Serum creatinine
SPSS: Statistical Product and Service Solutions
TNF-α: Tumor necrosis factor-alpha
